# Impairment of Cardiac Autonomic Nerve Function in Pre-school Children With Intractable Epilepsy

**DOI:** 10.3389/fneur.2021.632370

**Published:** 2021-06-25

**Authors:** Zhao Yang, Tung-Yang Cheng, Jin Deng, Zhiyan Wang, Xiaoya Qin, Xi Fang, Yuan Yuan, Hongwei Hao, Yuwu Jiang, Jianxiang Liao, Fei Yin, Yanhui Chen, Liping Zou, Baomin Li, Yuxing Gao, Xiaomei Shu, Shaoping Huang, Feng Gao, Jianmin Liang, Luming Li

**Affiliations:** ^1^National Engineering Laboratory for Neuromodulation, School of Aerospace Engineering, Tsinghua University, Beijing, China; ^2^Division of Pediatric Neurology, Pediatrics Department, Peking University First Hospital, Beijing, China; ^3^Department of Pediatric Epilepsy Center, Peking University First Hospital, Beijing, China; ^4^Department of Neurology, Shenzhen Children's Hospital, Shenzhen, China; ^5^Department of Pediatrics, Xiangya Hospital of Central South University, Changsha, China; ^6^Hunan Intellectual and Developmental Disabilities Research Center of Children, Changsha, China; ^7^Division of Pediatric Neurology, Pediatrics Department, Fujian Medical University Union Hospital, Fuzhou, China; ^8^Department of Epilepsy Center, Fujian Medical University Union Hospital, Fuzhou, China; ^9^Department of Pediatric, The People's Liberation Army (PLA) General Hospital, Beijing, China; ^10^Pediatics Department, Qilu Hospital of Shandong University, Jinan, China; ^11^Division of Pediatrics Neurology, Provincial Hospital Affiliated to Shandong University, Jinan, China; ^12^Department of Pediatrics, Affiliated Hospital of Zunyi Medical College, Zunyi, China; ^13^Department of Pediatrics, The Second Affiliated Hospital of Xi'an Jiaotong University, Xi'an, China; ^14^Department of Neurology, The Children's Hospital, ZheJiang University School of Medicine, Hangzhou, China; ^15^Department of Pediatric Neurology, First Bethune Hospital, Jilin University, Changchun, China; ^16^Research Center of Neuroscience, First Bethune Hospital, Jilin University, Changchun, China; ^17^Precision Medicine and Healthcare Research Center, Tsinghua-Berkeley Shenzhen Institute, Shenzhen, China; ^18^Institute of Human-Machine, School of Aerospace Engineering, Tsinghua University, Beijing, China; ^19^Center of Epilepsy, Beijing Institute for Brain Disorders, Beijing, China

**Keywords:** pre-school children, intractable epilepsy, heart rate variability, multiscale entropy, symbolization entropy

## Abstract

**Objective:** Intractable epilepsy and uncontrolled seizures could affect cardiac function and the autonomic nerve system with a negative impact on children's growth. The aim of this study was to investigate the variability and complexity of cardiac autonomic function in pre-school children with pediatric intractable epilepsy (PIE).

**Methods:** Twenty four-hour Holter electrocardiograms (ECGs) from 93 patients and 46 healthy control subjects aged 3–6 years were analyzed by the methods of traditional heart rate variability (HRV), multiscale entropy (MSE), and Kurths–Wessel symbolization entropy (KWSE). Receiver operating characteristic (ROC) curve analysis was used to estimate the overall discrimination ability. Net reclassification improvement (NRI) and integrated discrimination improvement (IDI) models were also analyzed.

**Results:** Pre-school children with PIE had significantly lower HRV measurements than healthy controls in time (Mean_RR, SDRR, RMSSD, pNN50) and frequency (VLF, LF, HF, LF/HF, TP) domains. For the MSE analysis, area 1_5 in awake state was lower, and areas 6_15 and 6_20 in sleep state were higher in PIE with a significant statistical difference. KWSE in the PIE group was also inferior to that in healthy controls. In ROC curve analysis, pNN50 had the greatest discriminatory power for PIE. Based on both NRI and IDI models, the combination of MSE indices (wake: area1_5 and sleep: area6_20) and KWSE (m = 2, τ = 1, α = 0.16) with traditional HRV measures had greater discriminatory power than any of the single HRV measures.

**Significance:** Impaired HRV and complexity were found in pre-school children with PIE. HRV, MSE, and KWSE could discriminate patients with PIE from subjects with normal cardiac complexity. These findings suggested that the MSE and KWSE methods may be helpful for assessing and understanding heart rate dynamics in younger children with epilepsy.

## Introduction

Epilepsy is a brain disorder affecting patients of all ages (1) with approximately 10.5 million children in the world suffering from uncontrolled seizures (1, 2), and 20–30% of these children are resistant to antiepileptic drugs (AEDs) and other clinical therapies (3). Sudden unexpected death in epilepsy (SUDEP) has been reported to account for 15 and 50% of all deaths in patients with epilepsy and drug-resistant epilepsy, respectively (4), and the regulation of the autonomic nervous system (ANS) has been highlighted (5). Specifically, alterations of the sympathetic and parasympathetic systems resulting in cardiac arrhythmia, apnea, or cerebral electrical shutdown have been linked to SUDEP (6, 7). Recurrent seizures have higher negative impact on physical growth, sleep, behavior, and mental development (such as depression, anxiety, psychosis, suicide) later in life, bringing heavy burdens to families and society (2, 3, 8–13). Apart from recurrent and unprovoked seizures, epilepsy also contributes to alterations of cardiac autonomic modulation, exhibiting an impairment of sympathetic and/or parasympathetic modulation of cardiac activity (14, 15). Children with intractable epilepsy demonstrate age-related seizure expression (2); thus, more attention should be paid to younger children, especially pre-school children, for their better future development. However, few studies have focused on the cardiac autonomic nerve system (CANS) of pre-school children with intractable epilepsy, and whether epilepsy and seizures impair the function of CANS in such younger age range is still unknown.

Heart rate variability (HRV) is considered one of the accurate biomarkers of the sympathovagal balance of the CANS by a noninvasive method. Generally, a high HRV reflects the sympathovagal balance or well-conditioned adaptability of CANS, and a low HRV relates to a sign of deficient and abnormal function of the ANS (16). HRV measurements have been effective and independent predictors for cardiovascular and neurological diseases (17, 18). Previous studies of autonomic modulation in children with epilepsy published different results, most of which indicate the impairment of CANS regulation considering HRV measurements in the time and frequency domain (14, 15, 19–26). In addition to 24-h long-term analysis, recent studies also focused on ictal or peri-ictal characters with the HRV method to investigate heart activity abnormalities or detect seizures (27–30). The inconsistency in the results, however, was probably owing to different designs of the experiments in sample size, epilepsy type, recording time, and analysis detail. Moreover, HRV index from 24-h Holter electrocardiogram (ECG) recording in pre-school children was still not covered in patients with intractable epilepsy and their healthy control subjects.

Heart rate signals have typical non-linear features because they are the results of the interaction of multiple physiological systems and are influenced by various internal and external factors (18). There are some limitations for traditional non-linear domain HRV measures to assess the complexity of heart rate dynamics (18). For example, classical entropy-based complexity measures quantify only the regularity of time series on a single scale without considering more scales from interaction and consolidative capability of time and space in CANS. Under the postulation that the healthy allow for responding to transient stressors for adaptation to the demands of an ever-changing environment, the multiscale entropy (MSE) method was proposed (31, 32). Diseased and/or aged systems are less adaptable, so the complexity of the human body should be reduced, which could be observed by MSE analysis but could not be observed using traditional HRV entropy. MSE has been extensively used and developed in diagnostics, classification, risk stratification, and prognosis for patients undergoing peritoneal dialysis as well as patients with diseases such as stroke, heart failure, primary aldosteronism, Alzheimer's disease, autism spectrum disorder, and Parkinson disease. Actually, MSE analysis is a kind of direct and fixed coarsening of RR intervals. Besides this, symbolization entropy is also a normal coarsening process to time series with adjustable control parameters for observing the effects of parameters and choosing the stable parameters. Kurths–Wessel symbolization entropy (KWSE) is a relatively easy symbolization method to implement and is closely related to heart rate signals, finally forming a four-symbol static time series transformation method (scope of recommendations for α: [0.03, 0.07] in original papers) (33–35). It has been used in the discrimination of elder, cardiac heart failure, and adult epilepsy from healthy people and seems to be a stable and reliable marker for cardiac complexity (36). However, MSE and KWSE analyses of heart rhythm dynamics in pre-school patients with intractable epilepsy have not yet been studied.

In our previous study, we looked into the difference of HRV and MSE features between adult patients with intractable epilepsy and healthy controls (37). Several indicators were found to have significant results. In this study, we aimed to investigate the variability and complexity of long-term ECG signals using not only HRV and MSE analysis, but also KWSE analysis in pre-school children with intractable epilepsy. Furthermore, the results of MSE and KWSE analysis were compared with traditional complexity measures.

## Materials and Methods

### Participants

Pediatric patients with intractable epilepsy (PIE) as defined by the International League Against Epilepsy (38) were screened strictly based on the inclusion and exclusion criteria in the VNS-PIE clinical trial. A total of 11 centers participated in the VNS-PIE study. These included Peking University First Hospital (Principal Unit), Chinese PLA General Hospital, Shenzhen Children's Hospital, Qilu Hospital of Shandong University, Shandong Province Hospital, the First Hospital of Jilin University, the Second Affiliated Hospital of Xi'an Jiaotong University, Xiangya Hospital Centeral South University, the Children's Hospital Zhejiang University School of Medical, Fujian Medical University Union Hospital, and the Affiliated Hospital of Zunyi Medical College. Pediatric patients had undergone routine pre-surgical examinations, including clinical history, biochemical examination, long term video-electroencephalograph (EEG), imaging examination (magnetic resonance or MR), cognitive development testing (Gesell scale), and 24-h Holter ECG recordings. Inclusion criteria were as follows (1) age 3–6 years, (2) at least six seizures per month, (3) refractory epilepsy, (4) in good health except for epilepsy, (5) family members of patients can understand the method and sign the informed consent, and (6) patients with good compliance and can complete post-operative follow-up. Exclusion criteria were as follows: (1) results of MRI showed epilepsy was caused by intracranial space-occupying lesions; (2) tumor, cardiopulmonary anomaly, heart failure, progressive neurological diseases, asthma, mental disease, peptic ulcer, diabetes, poor health, and other contraindications toward surgery; (3) vagal nerve lesion or damage; (4) could not write the epilepsy diary; (5) participating in another clinical trial; (6) could not complete the operation; (7) could not complete the post-operative follow-up; or (8) could not complete the programming. Pediatric healthy control (PHC) subjects with matched age and gender were recruited into this study based on their clinical history, physical examination results, and 24-h Holter ECG results. This trial (VNS-PIE) was approved by the Clinical Trial Ethics Committee of Peking University First Hospital (Protocol Number: G112L31101; Date: 31/7/2017) and registered on ClinicalTrials.gov protocol system (Clinical Trials Identifier: NCT03062514). The parents of all the pediatric participants provided informed consent in written form before the start of the study. The observed variables of participants included demographic data, seizure type, epilepsy duration, etiology, seizure frequency, number of AEDs used, pre-surgical MRI findings, ictal scalp video-EEG characteristic and ECG recordings.

### ECG Recording and Pre-processing

A 12-lead ambulatory ECG monitoring device (MIC-12H-3S; JincoMed, Beijing, China) with a sampling rate of 500 samples/s per channel was used to record a consecutive 24-h ECG in all participants. Wearing this Holter ECG device, participants (patients and healthy controls) were in freely moving conditions and normal daily style to avoid strenuous activities or restricted movement. Their parents were asked to keep a record of the children's main activity and observed seizures every hour, including the time, duration, and type of each activity and seizure. The chest lead V5 with stable and reliable signal quality was selected as the main analysis lead and the standard limb lead II as the secondary analysis lead. If the above two lead signals were missing or their signal-to-noise ratios were low, we selected other lead as an auxiliary analysis lead, which was recorded as a normal and stable waveform. The ECG segments with possible seizures along with the segments within at least 15 min before and from seizure onsets were discarded to avoid their potential effects on further analyses.

Based on Matlab (R2020a, Mathworks, Natick, MA, USA) and Kubios (Kubios Premium 3.4.1, University of Eastern Finland, Kuopio) softwares, the ECG fragments and abnormal QRS waves submerged by noise or motion artifacts were removed. The long-term RR interval time series were formed, whose abnormal R-wave markers were <10%, and the length of each record was not <20 h. Then 4-h periods of RR intervals in the awake and sleep state were selected, respectively, by researchers for each recording according to the heart rate characteristics and activity recordings (39, 40).

### Measures From RR Intervals

Traditional HRV measures always include time and frequency domain analysis according to the recommendations of the European Society of Cardiology and the North American Society of Pacing Electrophysiology (18). Mean RR was the mean RR interval values. SDRR was calculated as the standard deviation of RR intervals and taken to represent the overall variability of autonomic modulation. RMSSD was the root mean square of successive differences between successive RR intervals. pNN50 was calculated as the percentage of absolute differences in normal RR intervals >50 ms. RMSSD and pNN50 were regarded as the variability of parasympathetic nerve function on the heart rate. The frequency domain parameters, including high frequency (HF; 0.15–0.4 Hz), low frequency (LF; 0.04–0.15 Hz), and very low frequency (VLF; 0.003–0.04 Hz) power, were calculated by Fast Fourier transformation (FFT) algorithm. The total power (TP) was the sum of HF, LF, and VLF power, and the ratio of LF to HF (LF/HF) was also calculated. LF was taken to reflect the modulations of both the parasympathetic and sympathetic nervous systems, whereas HF primarily demonstrated the function of parasympathetic nerve. It was suggested that the VLF power appeared to be generated intrinsically by the heart itself (41).

Traditional non-linear HRV measures included ApEn (42) and SampEn (43) in this study. Instead of merely estimating the complexity of a time series with a single scale, the MSE method presents the meaningful structural richness of information embedded in multiple spatial and temporal scales (31, 32). In the analysis of MSE, we selected two 4-h periods of RR intervals in the quiet awake state and sleep state to reduce the variability of the circadian rhythm and physical activity. Similar processing can be found in previous research (37, 39, 40). The MSE method comprises two steps: (1) Coarse-graining process: the RR intervals were reconstructed as different time scales. For example, for a given RR interval, in which *N* is the number of time series, the multiple coarse-graining time series $y_{j}^{\left(\tau \right)}$ was the average of *n* non-overlapping consecutive beats with an increasing scale factor τ.The equations were calculated according to Equation (1):

(1) $$\begin{array}{r} y_{j}^{\left(\tau \right)}=\frac{1}{\tau }\overset{j\tau }{\underset{i=\left(j-1\right)\tau +1}{\sum }}RR_{i},\;1\leq j\leq N/\tau \end{array}$$

(2) Quantified by sample entropy with parameters *m* = 2 and *r* = 0.15 ^*^ SDRR, where *m* was the embedding dimension, ***r***was the size of the cell for coarse-graining the phase space, and SDRR was the standard deviation of the 4-h RR interval time series as defined in the paper in which the method was originally proposed (31, 32). The implementation of the parameters corresponded to normalizing the time series so that the sample entropy results depended only on the sequential ordering rather than the variance of the original time series. For each of the 4-h periods, including periods in the awake and sleep states, four different measures were calculated from the MSE profile: the area of entropy values of scales 1–5 (area 1_5), which quantified the complexity of RR dynamics in a short time scale; 6–15 (area 6_15) and 6–20 (area 6_20), which quantified the complexity of RR dynamics in long time scales; and the linear-fitted slope of scale 1–5 (k1), which characterized the modulation pattern in short scales (37, 39, 40). Such indices were proved to be efficient in our previous study discriminating adult patients with epilepsy and healthy controls (37). To avoid underestimation of entropy due to nonstationary artifacts, especially trends of RR intervals series, a detrending process was used on RR intervals before MSE analysis to adaptively extract the trends and subtract them (44, 45).

Besides the coarse graining on different scales in the MSE method, the symbolization of time series was also a coarse process by transforming the original sequence into a sequence containing only individual values, which showed fast, antinoise, and robust features in practical application. A static time series transformation method of four symbols determined KWSE with three steps (33–35). (1) In symbolization, the RR intervals were transformed to a symbolization series _*S*_*i*_(*RRi*_) based on Equation (2):

(2) $$S_{i}(RR_{i})=\{\begin{array}{c} 0:\;\mu <RR_{i}\leq (1+\alpha )\mu \\ \begin{array}{c} 1:\;(1+\alpha )\mu <RR_{i}\leq \infty \\ \begin{array}{c} 2:\;(1-\alpha )\mu <RR_{i}\leq \mu \;\\ 3:\;0<RR_{i}\leq (1-\alpha )\mu \; \end{array} \end{array} \end{array},\;1\leq i\leq N$$

where μ was the mean of the RR intervals series and α was the parameter to control the symbolization range. (2) Coding for the symbolization series, *C*_*i*_ was constructed by *m* points with τ delay based on Equation (3):

(3) $$\begin{array}{r} C_{i}=\overset{m}{\underset{j=1}{\sum }}{2^{m+1-j}S}_{i+\left(j-1\right)\tau },\;1\leq i\leq N-m\tau \end{array}$$

(3) With the Shannon entropy calculation, *H* was finally calculated by a classical Shannon entropy of *C*_*i*_ denoting the complexity of RR intervals based on Equation (4):

(4) $$\begin{array}{r} H=-\sum C_{i}C_{i} \end{array}$$

### Statistical Analysis

Continuous variables were presented as mean ± standard deviation (SD). Comparisons of continuous data between the PIE and control groups were made using the Mann–Whitney *U*-test. Differences between qualitative or categorical variables were assessed using the chi-square test or Fisher's exact test. To compare the ability of different Holter parameters to differentiate the PIE patients from the healthy control patients, a receiver operating characteristic curve (ROC) was constructed from the sensitivity and specificity with logistic regression models, and the area under the ROC curve (AUC) was used to estimate the overall discrimination ability. C-statistics were used to describe the discrimination of the models before and after adding non-linear parameters (46–48). Net reclassification improvement (NRI) and integrated discrimination improvement (IDI) were used to assess improvement of the discriminating power by using two different logistic regression models with 0.2 and 0.4 as the cutoff points, which are commonly used cutoff values (46, 48). All statistical analyses were performed using SPSS (Version 20, IBM Corp., Armonk, NY, USA) and Matlab (R2020a, Mathworks, Natick, MA, USA). Statistical significance was set at *p* <0.05.

## Results

### Demographics and Clinical Factors

A total of 93 patients with PIE and 46 healthy control participants were included in this study, according to the protocols. Among the 93 patients, based on their epileptic diary, 25 were reported to have possible seizures during the 24-h ECG recording, and 21 reported to have focal seizures lasting <60 s.

Demographic data and clinical factors of patients with PIE (*n* = 93, range 3.1–5.6 years old) and healthy controls (*n* = 46, range 3.0 to 5.5 years old) are presented in Table 1. Demographic data, including gender, age, and body mass index (BMI), showed no significant differences between patients and controls. However, there were significant statistical differences in each subitem of the Gesell scale, indicating that patients with PIE were undergoing general heavy mental and cognitive degradation. Other clinical factors included epilepsy duration, use of AEDs, seizure frequency, seizure type, and cerebral lesions. Twelve types of therapeutic AEDs were reported to be previously used, among which valproate was the most administered one, taken by 72 subjects. Detailed information of clinical and therapeutic characteristics of patients are also shown in Table 1.

**Table 1:** Demographic data and clinical data of the patients.

**Characteristics**	**PIE** ** (*n* = 93)**	**PHC** ** (*n* = 46)**	***P*-value**
**Male, no. (%)**	60 (64.5)	27 (58.7)	0.50
**Age (years)**	4.5 ± 0.8	4.2 ± 0.8	0.09
**Body mass index (kg/m**^**2**^**)**	15.9 ± 2.1	15.6 ± 1.9	0.31
**Gesell scale**			
Adaptability	25.5 ± 21.1	97.5 ± 7.3	<0.001
Gross motor	30.8 ± 19.4	93.7 ± 9.4	<0.001
Fine motor	27.2 ± 21.9	95.5 ± 7.2	<0.001
Language	24.6 ± 20.1	96.4 ± 9.5	<0.001
Individual and social interaction	26.9 ± 21.4	97.2 ± 9.5	<0.001
**Epilepsy duration (months)**	39.7 ± 13.4	N.A.	N.A.
**No. of previous AEDs**	5.4 ± 2.1	N.A.	N.A.
**No. of present AEDs**	2.9 ± 1.2	N.A.	N.A.
**Seizures per month**	370.3 ± 488.6	N.A.	N.A.
**Seizure type, no. (%)**			
Tonic seizure	38 (40.9)	N.A.	N.A.
Atypical absence	10 (10.8)	N.A.	N.A.
Atonic seizure	6 (6.5)	N.A.	N.A.
Myoclonic seizure	17 (18.3)	N.A.	N.A.
Spasm	45 (48.4)	N.A.	N.A.
Others	20 (21.5)	N.A.	N.A.
**Cerebral lesions (MRI), no. (%)**			
Temporal	2 (2.2)	N.A.	N.A.
Frontal	7 (7.5)	N.A.	N.A.
Parietal	12 (12.9)	N.A.	N.A.
Occipital	11 (11.8)	N.A.	N.A.
Diffusing or multiple lesions	10 (10.8)	N.A.	N.A.
Negative	57 (61.3)	N.A.	N.A.
**Previous usage of AEDs, no. (%)**			
Valproate	72 (77.4)	N.A.	N.A.
Topiramate	48 (51.6)	N.A.	N.A.
Levetiracetam	39 (41.9)	N.A.	N.A.
Clonazepam	33 (35.5)	N.A.	N.A.
Lamotrigine	24 (25.8)	N.A.	N.A.
Oxcarbazepine	16 (17.2)	N.A.	N.A.
Clobazam	15 (16.1)	N.A.	N.A.
Zonisamide	5 (5.4)	N.A.	N.A.
Vigabatrin	5 (5.4)	N.A.	N.A.
Carbamazepine	3 (3.2)	N.A.	N.A.
Phenobarbital	2 (2.2)	N.A.	N.A.
Rufinamide	1 (1.1)	N.A.	N.A.

*Data were presented as mean value ± standard deviation or number (percentage). No. denotes “number.” N.A. denotes “not available,” and five patients' BMI were not available*.

### ECG Signals Analysis

The measurements of traditional HRV analysis including time and frequency domain from long-term RR intervals were significantly lower in patients with PIE than that in healthy controls, whereas non-linear parameters (ApEn and SampEn) could not differentiate the two populations (Table 2). The result imply impairment of the function of CANS in PIE patients compared with healthy controls as expected.

**Table 2:** Traditional HRV and MSE measurements of PIE and PHC participants.

**Measures**	**PIE** ** (*n* = 93)**	**PHC** ** (*n* = 46)**	***P*-value**
**Mean_RR(ms)**	580.4 ± 52.8	616.4 ± 58.7	0.003
**SDRR(ms)**	33.6 ± 21.8	45.1 ± 15.9	<0.001
**RMSSD(ms)**	36.7 ± 25.7	50.6 ± 23.3	<0.001
**pNN50(%)**	9.0 ± 8.8	19.0 ± 11.4	<0.001
**VLF(ms**^**2**^**)**	105.3 ± 169.7	155.7 ± 77.3	<0.001
**LF(ms**^**2**^**)**	482.8 ± 512.4	815.0 ± 452.4	<0.001
**HF(ms**^**2**^**)**	714.4 ± 865.9	1428.9 ± 1235.3	<0.001
**LF/HF(ms**^**2**^**)**	1.1 ± 0.8	0.8 ± 0.3	0.04
**TP(ms**^**2**^**)**	1304.4 ± 1406.2	2401.7 ± 1683.5	<0.001
**ApEn**	1.42 ± 0.07	1.42 ± 0.05	0.58
**SampEn**	1.55 ± 0.15	1.53 ± 0.10	0.42
**MSE**			
**Wake**			
area1_5	4.43 ± 0.97	4.93 ± 1.05	0.02
area6_15	12.88 ± 2.30	13.75 ± 1.95	0.05
area6_20	20.41 ± 3.60	21.62 ± 2.98	0.08
k1	0.05 ± 0.06	0.06 ± 0.06	0.35
**Sleep**			
area1_5	5.34 ± 0.93	5.39 ± 0.95	0.66
area6_15	11.94 ± 2.01	11.09 ± 1.95	0.02
area6_20	18.48 ± 3.26	16.74 ± 3.09	0.003
k1	−0.05 ± 0.07	−0.07 ± 0.06	0.08

*Data were presented as mean value ± standard deviation*.

**Figure 1:**
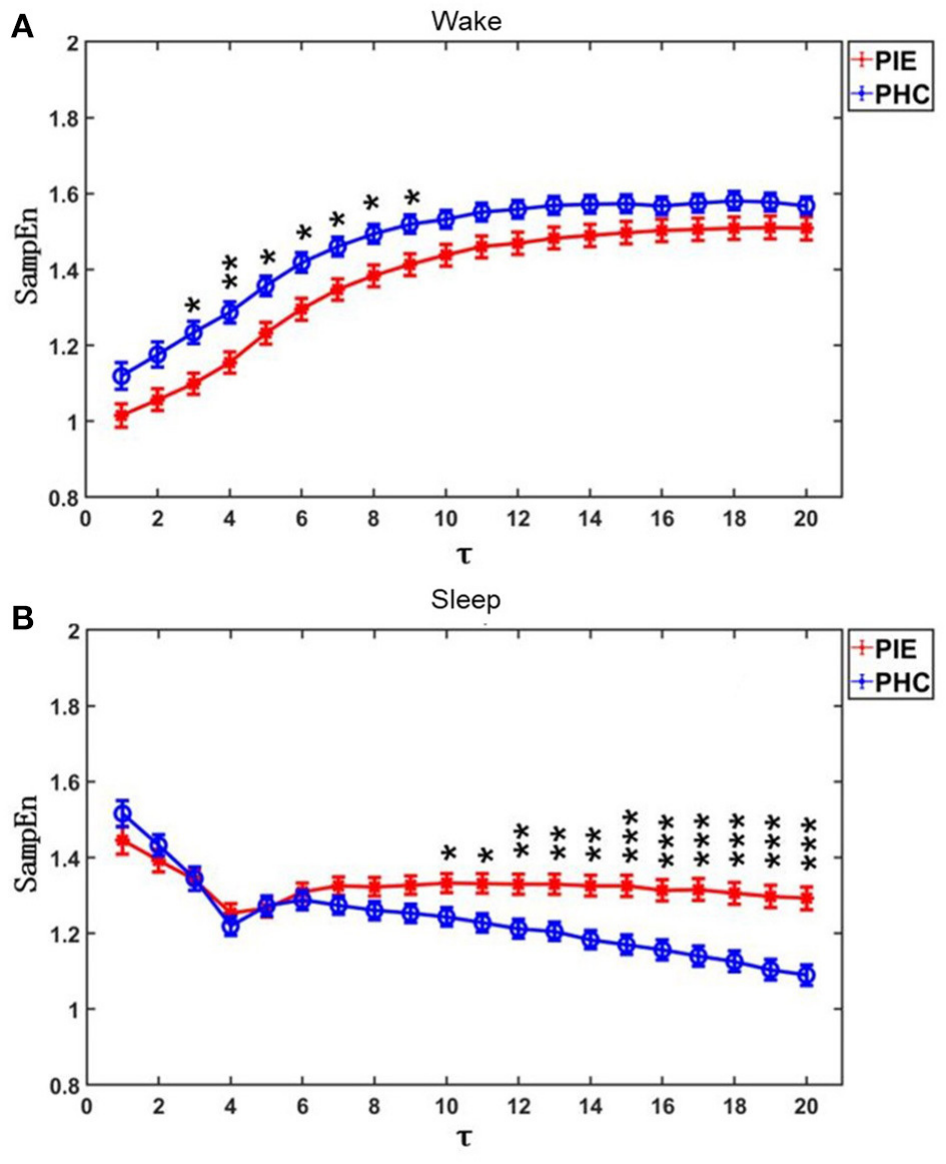
MSE profiles for PIE and PHC. Data are shown as mean value and bars denoted the standard error ($SE=SD/\sqrt{n}$). **(A)** Participants were on wake state; **(B)** Participants were on sleep state. τ was the scales. *P*-value was calculated by Mann-Whitney *U*-test. *p < 0.05; **p < 0.01; ***p < 0.001.

The results of MSE are shown in Figure 1 and Table 2. The profiles of MSE were different based on the wake/sleep state, which might show the influence of circadian rhythm and the state of the brain. In comparison to pediatric healthy controls, we found the MSE of PIE patients in a scale of 3–9 in the wake state to be significantly lower as well as scale 10–20 in the sleep state significantly higher. Also, we found that, in PIE patients, area 1_5 in the wake state was significantly lower and areas 6_15 and 6_20 in the sleep state were significantly higher than the PHC group. No significant differences were found in other measures of MSE. The significant results show the potential discriminating power of MSE measures between PIE patients and PHC, indicating the impairment in the complexity of CANS in PIE patients. Although, as we can observe from the results, some of the measures of MSE might not work as efficiently as traditional time and frequency domain HRV analysis to distinguish PIE and PHC participants.

**Figure 2:**
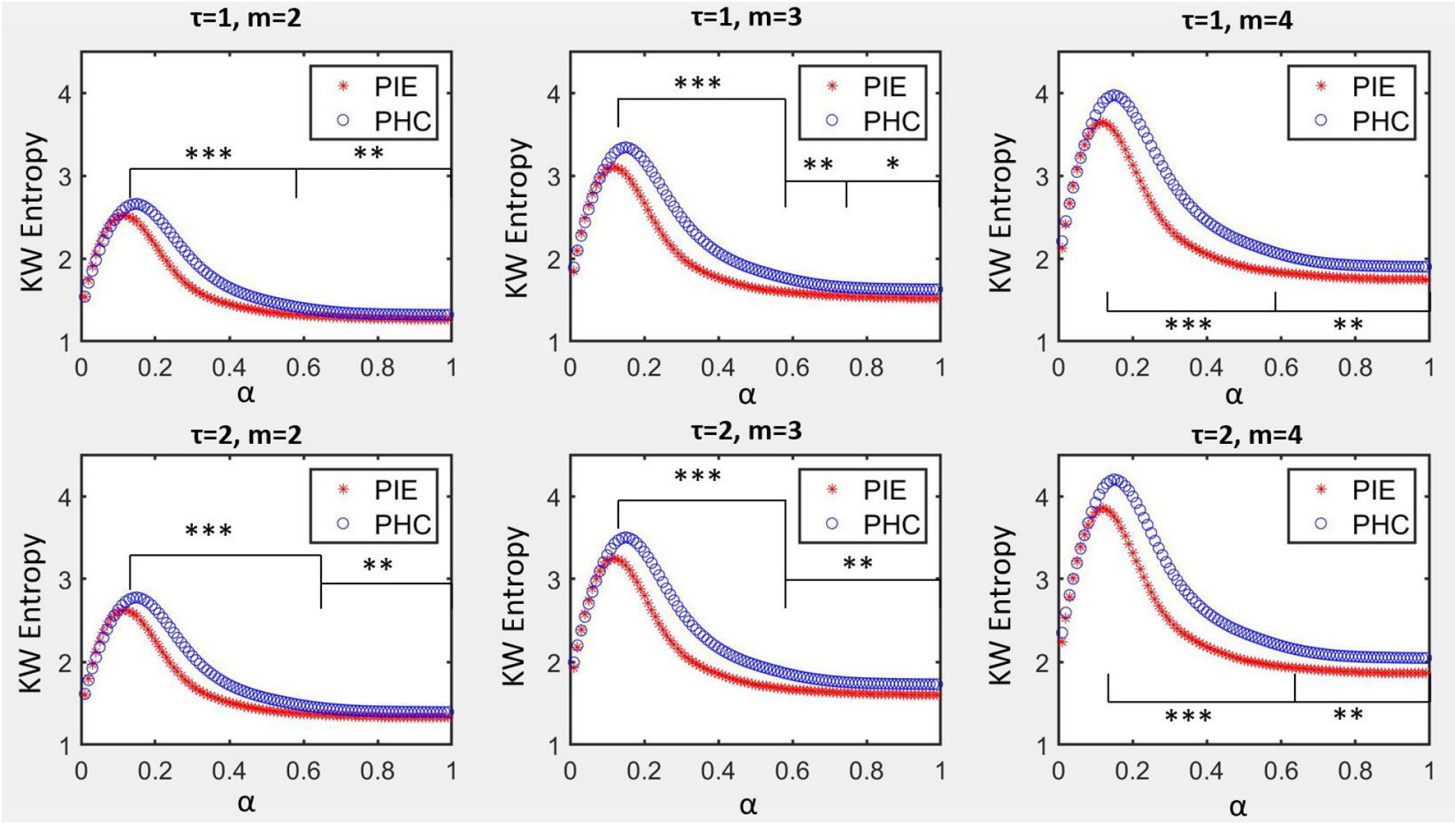
KW entropy profiles for PIE and PHC. Data are shown as mean value. α was the parameter to control the symbolization range for symbolization series *S*_*i*_, m was the size of word of coded series *C*_*i*_, τ was the delay of coded series *C*_*i*_. *p < 0.05; **p < 0.01; ***p < 0.001.

The profile of KWSE is presented in Figure 2. The larger the *m* parameter, the higher the amplitude of the total KWSE curve. Also, no matter which of the six combinations of *m* (2, 3, 4) and τ (1, 2) selected, the entropy values between the two groups were significantly different when α ≥ 0.12, and especially, the *p*-value did not exceed 0.001 when α was between 0.14 and 0.61.

From the results above, we can also find that, in quantifying the complexity of the heart rate signals, the MSE and KWSE worked better than the traditional HRV non-linear parameters ApEn and SampEn. This provided evidence of potentially higher discriminating power when traditional HRV and MSE/KWSE are combined during the modeling.

### ROC Curve Analysis

**Figure 3:**
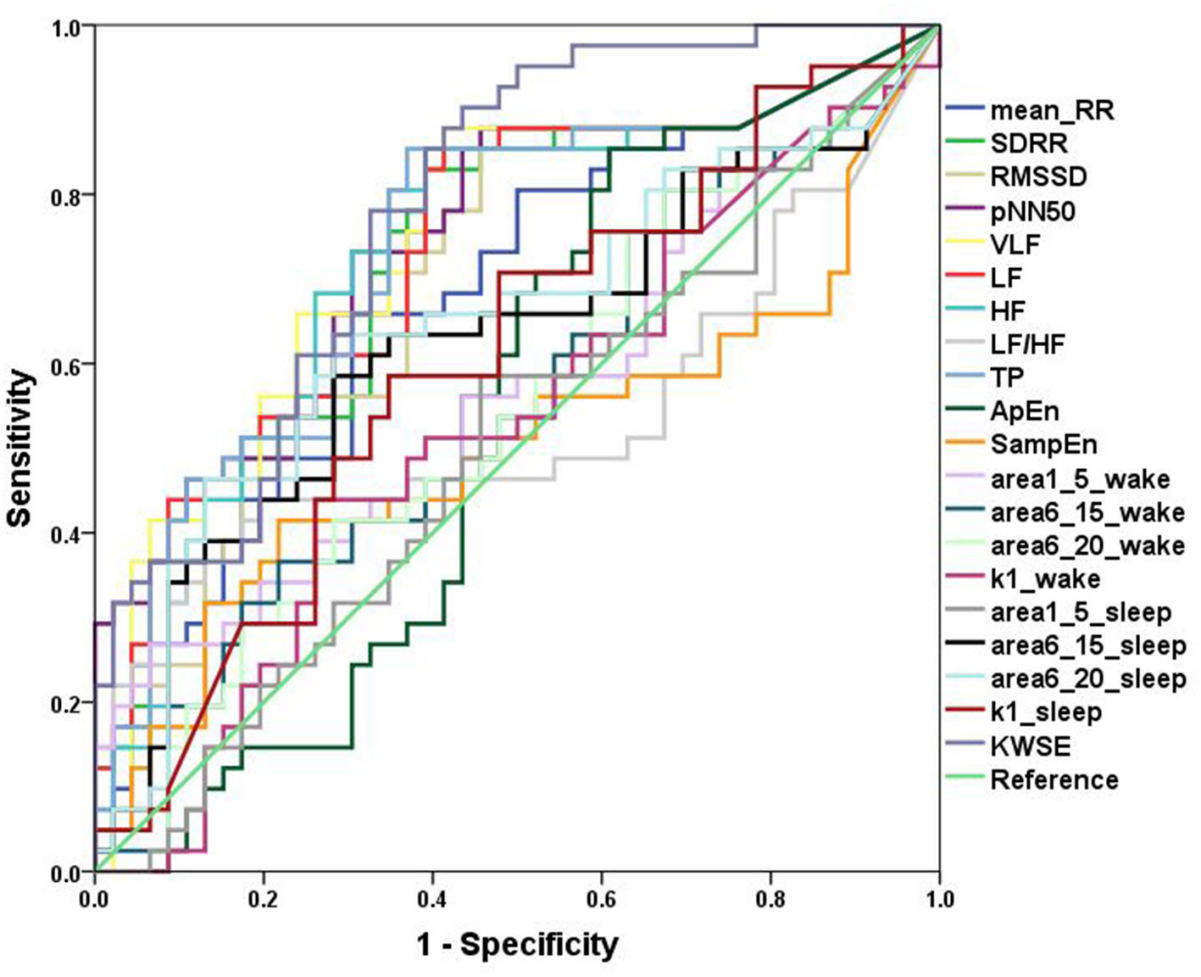
ROC curves. Analysis of the discrimination power of the two groups by ROC curve analysis. TheAUC of Mean_RR, SDRR, RMSSD, pNN50, VLF, LF, HF, LF/HF, TP, ApEn, SampEn, area1_5_wake, area6_15_wake, area6_20_wake, k1_wake, area1_5_sleep, area6_15_ sleep, area6_20_ sleep, k1_ sleep, KWSE (m = 2, τ = 1, α = 0.16) were 0.680, 0.727, 0.706, 0.746, 0.743, 0.734, 0.732, 0.531, 0.736, 0.536,0.505, 0.568, 0.550, 0.546, 0.528, 0.504, 0.624, 0.648, 0.603, and 0.783, respectively.

In the ROC curve analysis of a single variable, for traditional HRV parameters, pNN50 (AUC = 0.746) had the greatest discriminatory power for patients with PIE and healthy control subjects and complexity measures (Figure 3). In quantifying the complexity, the AUC of non-linear HRV parameters ApEn and SampEn were 0.536 and 0.505, respectively. For the MSE results, in the wake state, the AUC of area1_5, area6_15, area6_20, and k1 were 0.568, 0.550, 0.546, and 0.528, respectively, and in the sleep state, the AUC of area1_5, area6_15, area6_20, and k1 were 0.504, 0.624, 0.648, and 0.603, respectively. The result of KWSE (*m* = 2, τ = 1, α = 0.16) was 0.783, which had the maximum distinguishing ability. This again showed that the MSE measures and KWSE overall had more discriminating power than the non-linear HRV when quantifying the complexity of the RR time series.

Then, we looked at the combination of one of the traditional HRV parameters and one of the heart rate complexity measures (MSE or KWSE). In this analysis, according to the profiles of MSE and KWSE exhibited in section ECG Signals Analysis, we selected MSE indices (wake: area1_5 and sleep: area6_20) and KWSE (*m* = 2, τ = 1, α = 0.16) to construct the model. As a result, we found remarkable improvement in the discriminating power as shown in Table 3. The results show that all of the AUCs of the combined models for each of the traditional HRV measure are over 0.783, larger than that of any single HRV measure alone. Among them, the HRV adding KWSE models always had larger AUC than that adding MSE models with the largest being 0.861 of pNN50+KWSE. In both the NRI and IDI indices, the results confirm that the combination of the traditional HRV and MSE/KWSE had greater discriminatory power than any of the single HRV measures with all the NRI and IDI values positive, and all *p*-values <0.05. This result proved that the combination of the traditional HRV and MSE/KWSE was more efficient than single variable models, and the HRV+KWSE models seemed to be the most promising indices for the improvement of discrimination.

**Table 3:** AUC, NRI, and IDI models of traditional HRV parameters adding heart rate complexity parameters.

**Measures**		**AUC**	**R square**	**NRI**	**NRI:*p*-value**	**IDI**	**IDI:*p*-value**
SDRR		0.727	0.273				
	Wake:area1_5	0.781	0.418	0.451	0.002	0.142	0.001
	Sleep:area6_20	0.798	0.425	0.452	0.002	0.146	0.001
	KWSE^*^	0.814	0.428	0.488	0.001	0.152	0.001
RMSSD		0.706	0.226				
	Wake:area1_5	0.802	0.402	0.387	0.006	0.143	0.002
	Sleep:area6_20	0.798	0.394	0.409	0.003	0.107	0.009
	KWSE^*^	0.828	0.427	0.481	0.001	0.142	0.001
pNN50		0.746	0.234				
	Wake:area1_5	0.829	0.432	0.562	<0.001	0.155	<0.001
	Sleep:area6_20	0.831	0.432	0.568	<0.001	0.159	<0.001
	KWSE^*^	0.861	0.455	0.604	<0.001	0.167	<0.001
VLF		0.743	0.231				
	Wake:area1_5	0.812	0.421	0.543	<0.001	0.147	<0.001
	Sleep:area6_20	0.814	0.423	0.567	<0.001	0.139	0.001
	KWSE^*^	0.836	0.439	0.573	<0.001	0.161	<0.001
LF		0.734	0.229				
	Wake:area1_5	0.825	0.428	0.341	0.004	0.122	0.004
	Sleep:area6_20	0.832	0.429	0.419	0.002	0.125	0.004
	KWSE^*^	0.841	0.443	0.498	0.001	0.149	0.001
HF		0.732	0.228				
	Wake:area1_5	0.819	0.417	0.507	<0.001	0.156	0.001
	Sleep:area6_20	0.823	0.424	0.517	<0.001	0.154	0.001
	KWSE^*^	0.843	0.429	0.533	<0.001	0.161	0.001
TP		0.736	0.231				
	Wake:area1_5	0.828	0.431	0.541	<0.001	0.162	<0.001
	Sleep:area6_20	0.825	0.428	0.531	<0.001	0.161	<0.001
	KWSE^*^	0.839	0.439	0.587	<0.001	0.185	<0.001

*AUC, areas under the curve; NRI, net reclassification improvement; IDI, integrated discrimination improvement; MSE, multiscale entropy. KWSE, Kurths–Wessel symbol entropy*.

* *m = 2, τ = 1, α = 0.16*.

## Discussion

The main findings of this study were (1) the pre-school children with PIE had worse heart rhythm complexity than age- and gender-matched healthy control children, which was first studied to the best of our knowledge; (2) in all linear and non-linear measures based on heart rate, pNN50, VLF, and KWSE had the greatest discriminatory power to detect the patients undergoing PIE as a single parameter, and these were superior to the traditional non-linear measures; (3) the combination of traditional HRV measures and MSE/KWSE increased the discriminatory power to differentiate PIE from healthy controls, and the HRV+KWSE models had the most promising results. The demographics and clinical factors did not demonstrate significant impact on our results.

Traditional time and frequency domain analysis of HRV is a conventional and useful tool to evaluate the cardiac autonomic system and is commonly used to stratify the risk of patients with cardiovascular and neurological diseases (17, 18). Aging and disease have long been the main factors to be focused on for dynamic characteristics of heart rate (18). According to our results, we can not only find a major degradation in behavior and cognition based on the Gesell scale, but also observe a general impairment of the cardiac autonomic function by analyzing the RR time series. By the method of traditional HRV, the values of the time and frequency domain parameters of the pre-school PIE patients, including SDRR, RMSSD, pNN50, VLF, LF, and HF, are significantly lower than the paired healthy controls, reflecting prominent autonomic dysfunction in both the sympathetic and the vagal tone. This result is consistent with most of the previous studies on CANS functions of children with epilepsy, which indicate the impairment of CANS regulation with multiple decrease in HRV measurements in the time and frequency domain, such as HF, LF, RMSSD, and pNN50 (17, 19, 20, 22). We also noticed that the LF/HF result of the PIE group was significantly higher than the PHC group, indicating the imbalance of the CANS in patients. Several studies as well found the imbalance of sympathetic vagal with LF increase or LF/HF increase (23, 26, 49). However, some studies found no alterations on HRV measures (14, 24). The difference of these results may be owing to inconsistent sample size, epilepsy type, recording time, and analysis detail and so on. Still, few studies concern pre-school children with PIE even in the healthy state. Our result first offers evidence of the impairment of cardiac autonomic function in epilepsy patients of pre-school age group, which supports the extension of the previous conclusion to a wider age range.

Non-linear features are important to characterize and quantify the dynamic variation of physiological systems including CANS (18). Apart from traditional non-linear domain measures of HRV, the MSE and KWSE methods were also included in our analysis, which consider more scales of temporal and spatial interaction in CANS (31–35). From our data, although traditional non-linear domain HRV ApEn and SampEn failed to reveal the decrement of the complexity in the patients, the MSE and KWSE methods, however, successfully proved this alteration. Several scales and indices of MSE and KWSE of the PIE group showed significant differences from the PHC group. The decrease of complexity under free-running conditions reflected a declined ability of the systems to function in certain dynamical regimes, possibly due to dysregulation or impairment of autonomic control mechanisms. The results demonstrate the dysfunction of CANS in PIE and, in the meantime, proved the efficiency of the multiscale methods compared with the traditional single-scale methods, which matched our previous expectation. However, no previous study has reported the results of MSE and KWSE analysis in pre-school children with PIE. Recent studies of MSE on pediatric patients with epilepsy focused mainly on the EEG signals, which all showed that healthy controls had more complexity than epilepsy patients (50–54). Our result complemented this conclusion with the ECG signals and might provide new insights into cardiac complexity in epilepsy.

In the modeling of the discrimination of the patients and healthy controls, we also found that models including multiscale measures worked better than any single-index model. The largest AUC of the models increased from 0.783 with HRV indices alone to 0.861 with HRV and MSE/KWSE combined. Among them, we found that the HRV+KWSE seemed to work the most efficiently. The results show the effectiveness of the MSE/KWSE as auxiliary methods in the modeling of discrimination. Particularly, the KWSE method seemed to be the most promising method for the improvement of the models for pre-school children with epilepsy. In addition to the results, we also chose the parameters carefully to get the dynamic features on the heart rate. The original recommendation range for α in analysis of KWSE was not suitable for pre-school children in our data, and the interval [0.12, 0.99] had more stable discrimination power. We selected the set of parameters as *m* = 2, τ = 1, α = 0.16, to simplify the calculation while preserving the discriminating power. In short, this is the first modeling of the discrimination of PIE patients and healthy controls of the pre-school age group based on ECG signals, and by the HRV with MSE/KWSE measures, the models exhibited enough efficiency.

There were also some limitations in our study: (1) Although the age range was focused and narrow, we recruited heterogeneous children with various epilepsy etiologies, epilepsy durations, kinds of AEDs, seizure frequency, seizure type, cerebral lesions, mental development, and so on. These factors may have potential impact on variability and complexity for CANS. For example, for studies focusing on specific syndromes, Hattori et al. (26) found that LF significantly improved from 10-min ECG in sleep state for children with West syndrome aged <1 year. Other studies focusing on Dravet syndrome found a total decline in multiple HRV indices on 24-h ECG (22, 55), and Delogu et al. (22) also found no significant results in all HRVs for patients with other syndromes. These results show that complicated factors that affect the results can be significant. (2) We only roughly deleted possible seizure episodes based on ECG data by visual inspection. Because the EEG data is considered as the gold standard of identification of the seizure episodes, there might remain undetected seizure episodes in our pre-processed ECG data. They might impact our results because some studies have published the effects of seizure episodes on heart rate and HRV (27–30). Further studies are needed to explore the impact of seizures on the CANS of pre-school children.

## Conclusion

PIE in pre-school children is associated with diminished HRV, MSE, and KWSE measures, thereby reflecting the loss of sympathetic vagal balance and function of autonomic system on heart rate. More importantly, when modeling with traditional HRV measurements, the combinations with MSE and KWSE significantly improve the power to differentiate PIE from healthy subjects. These quantification methods of HRV could also be used in younger children and may provide new insights into the cardiac complexity in epilepsy.

## Data Availability Statement

The raw data supporting the conclusions of this article will be made available by the authors, without undue reservation.

## Ethics Statement

The studies involving human participants were reviewed and approved by the Clinical Trial Ethics Committee of Peking University First Hospital. Written informed consent to participate in this study was provided by the participants' legal guardian/next of kin.

## Author Contributions

All authors listed have made a substantial, direct and intellectual contribution to the work, and approved it for publication.

## Conflict of Interest

HH and LL reported personal fees from Beijing Pins Medical Co., outside the submitted work. The funder was not involved in the study design, collection, analysis, interpretation of data, the writing of this article or the decision to submit it for publication. The remaining authors declare that the research was conducted in the absence of any commercial or financial relationships that could be construed as a potential conflict of interest.
